# Knowledge and attitudes of theatre staff prior to the implementation of robotic-assisted surgery in the public sector

**DOI:** 10.1371/journal.pone.0213840

**Published:** 2019-03-14

**Authors:** Kate E. McBride, Daniel Steffens, Kylie Duncan, Paul G. Bannon, Michael J. Solomon

**Affiliations:** 1 RPA Institute of Academic Surgery (IAS), Royal Prince Alfred Hospital and University of Sydney, Sydney, New South Wales, Australia; 2 Faculty of Medicine and Health, The University of Sydney, Sydney, New South Wales, Australia; 3 Surgical Outcomes Research Centre (SOuRCe), Royal Prince Alfred Hospital, Sydney, New South Wales, Australia; 4 The Baird Institute, Sydney, New South Wales, Australia; University of Alabama at Birmingham, UNITED STATES

## Abstract

**Background:**

The use of robotic-assisted surgery (RAS) is becoming increasingly prevalent across a range of surgical specialties within public hospitals around Australia. As a result, it is critical that organisations consider workplace factors such as staff knowledge, attitudes and behaviours prior to the implementation of such new technology. This study aimed to describe the knowledge and attitudes of operating theatre staff from a large public tertiary referral hospital prior to the commencement of an RAS program.

**Methods:**

A cross-sectional survey of nursing, medical and support staff working in the operating theatre complex of a large public tertiary referral hospital was completed over a one-week period in June 2016. A 23-item questionnaire was utilised for data collection.

**Results:**

164 (66%) theatre staff returned the surveys and were included in this study. The majority of medical staff reported being knowledgeable about RAS, whilst the majority of nursing and support staff did not. Overall the theatre staff were neutral about the potential benefits of RAS to patients. The majority of medical staff believed the implementation of RAS will increase the value of staff roles and job satisfaction, while nursing and support staff were uncertain about these benefits. All three staff groups were concerned about the impact of an RAS program on Workplace Health and Safety, and care and handling.

**Conclusion:**

Operating theatre staff presented different knowledge and attitudes prior to the introduction of RAS. Whilst theatre staff were more favourable towards RAS than negative, they largely reserved their judgement about the new system prior to their own experiences. Collectively, these findings should be taken into consideration for training and support strategies prior to the implementation of a RAS program.

## Introduction

Within the Australian healthcare system, robotic-assisted surgery (RAS) has largely been undertaken within the private sector. Since the first case was undertaken in Melbourne in 2003, there are now 43 da Vinci surgical systems (Si and Xi) currently in use in Australia with 36 (84%) of those based within private hospitals.[1] The use of RAS however is rapidly evolving and becoming increasingly prevalent across a range of surgical specialties within public hospital operating theatres around Australia.[2]

While the impact of RAS on the skills and training of surgeons and surgical trainees has been considered,[3–5] it is evident there are implications for the entire multidisciplinary theatre team. Certainly the safety and efficiency of an RAS program is dependent on the presence of a consistent theatre team that is proficient in the use of the technology.[6] The introduction of RAS brings potential change not only to the roles and workloads of staff within the perioperative environment, but also to their satisfaction, sense of value and involvement in the delivery of patient care.[6–8]

As a result, it is critical that organisations consider workplace factors such as staff culture, attitudes and behaviours prior to the implementation of new technology. This is particularly the case for RAS in the public sector where the level of staff exposure to the technology is likely to be lower. As such, this study aims to identify and describe the knowledge and attitudes of operating theatre staff from a large public tertiary referral hospital in Australia on RAS prior to the commencement of a new program involving complex intra-operative technology.

## Materials and methods

### Study design

This is a cross-sectional study that surveyed all staff working in the operating theatre complex of Royal Prince Alfred Hospital in Sydney over a one-week period in June 2016. All included participants provided their written informed consent and the Royal Prince Alfred Hospital ethics committee approved the study protocol (Protocol X16-0049 and LNR/16/RPAH/59).

### Participants

Theatre staff were categorised into three groups according to their specialty: (i) Nursing (included all nursing awards); (ii) Medical Staff (included consultant surgeon, consultant anaesthetist, fellow, surgical registrar, anaesthetics registrar, resident and intern); and (iii) Support Staff (included radiographer, operations assistant, administrative officer and theatre technician). Only staff that were rostered on during the study period were invited to participate.

### Study survey

The study survey consisted of two main sections. Section 1 captured basic demographics, including gender, age category, staff specialty, knowledge and skill level in the use of the da Vinci surgical system. Section 2 captured the potential benefits of RAS for patients (length of hospital stay, postoperative pain, and intraoperative complications), for staff (value of staff role, job satisfaction and enhancement of staff knowledge), Workplace Environment (work health and safety, care and handling, maintenance of sterile field, decrease in direct involvement, operation time, space and location, team dynamics, cost and financial pressure) and facilitators towards the implementation of new technology (theoretical and practical training, educational guides and staff support).

Participants were asked to rate their level of agreement with the abovementioned constructs according to the following responses: (i) agree; (ii) neutral; and (iii) disagree. Survey participation was voluntary and anonymous with no identifying information collected.

The study survey was based on the Research Capacity in Context Tool developed by Queensland Health and Griffith University.[9] The survey was pilot tested on 15 hospital staff to check for clarity of wording. The administered RAS survey can be found on S1 Survey.

### Data synthesis

Survey response data was manually entered and stored within REDCap database.[10] A basic descriptive analysis was performed according to theatre staff specialty (nursing staff, medical staff and support staff). Differences in responses between the three theatre staff specialties were calculated using Chi-square test. A significant p value was set as 0.05 for all analyses. All statistical analyses were performed using IBM SPSS Statistics version 24 (SPSS Inc., Chicago, IL, USA).

## Results

Of the 250 theatre staff that were invited to participate, 164 (66%) returned their survey and were included in this study (nursing staff = 77; medical staff = 47; and support staff = 40). Most of the responders were female (56.7%) and between 35 and 49 years of age (40.4%). Medical staff reported the highest rate of RAS knowledge (70.7%), while the support staff group reported the highest rate of robotic skills (23.4%). The characteristics of the study sample are reported in Table 1. The three specialty groups presented differences in terms of gender, age, robotic knowledge and robotic skills.

**Table 1:** Characteristics of the study sample categorised according to specialty.

Staff characteristics	Overall, n (%)	Nursing Staff, n (%)	Medical Staff, n (%)	Support Staff, n (%)	P value
**Gender**	**N = 164**	**N = 77**	**N = 47**	**N = 40**	
Male	71 (43.3)	12 (15.6)	38 (80.9)	21 (52.5)	<0.001
Female	93 (56.7)	65 (84.4)	9 (19.1)	19 (47.5)	
**Age, years**	**N = 161**	**N = 74**	**N = 47**	**N = 40**	
18 to 34	46 (28.6)	34 (45.9)	9 (19.1)	3 (7.5)	<0.001
35 to 49	65 (40.4)	27 (36.5)	24 (51.1)	14 (35.0)	
≥50	50 (31.1)	13 (17.6)	14 (29.8)	23 (57.5)	
**Robotic Knowledge**	**N = 152**	**N = 74**	**N = 41**	**N = 37**	
None	81 (53.3)	43 (58.1)	12 (29.3)	26 (70.3)	0.001
Some/advanced	71 (46.7)	31 (41.9)	29 (70.7)	11 (29.7)	
**Robotic Skill**	**N = 159**	**N = 75**	**N = 47**	**N = 37**	
None	135 (84.9)	71 (94.7)	36 (76.6)	28 (75.7)	0.005
Some/Advanced	24 (15.1)	4 (5.3)	11 (23.4)	9 (24.3)	

Medical staff: included consultant surgeon, consultant anaesthetist, fellow, surgical registrar, anaesthetics registrar, resident and intern. Support staff: included radiographer, operations assistant, administrative officer and theatre technician.

### Benefits to patients

The majority of theatre staff were neutral about the potential benefits of RAS to patients. Medical staff believed that RAS could reduce patients’ length of hospital stay (55.3%). However, there were differences between the three specialty groups towards the benefit of robotic surgery to patients (Table 2).

**Table 2:** Staff attitude towards the implementation of robotic surgery categorised according to specialty.

Staff attitude	Nursing Staff, n (%)			Medical Staff, n (%)			Support Staff, n (%)			P value
	Agree	Neutral	Disagree	Agree	Neutral	Disagree	Agree	Neutral	Disagree	
**Benefits to patients** (n = 162)										
Reduce length of hospital stay	34 (44.2)	**41 (53.2)**	2 (2.6)	**26 (55.3)**	13 (27.7)	8 (17.0)	11 (28.9)	**26 (68.4)**	1 (2.6)	<0.001
Less postoperative pain	26 (33.8)	**49 (63.6)**	2 (2.6)	19 (40.4)	**21 (44.7)**	7 (14.9)	11 (28.9)	**26 (68.4)**	1 (2.6)	0.022
Less intraoperative complications	23 (29.9)	**51 (66.2)**	3 (3.9)	7 (14.9)	**31 (66.0)**	9 (19.1)	15 (39.5)	**22 (57.9)**	1 (2.6)	0.004
**Benefits to Staff** (n = 160)										
Increase value of staff role	25 (32.9)	**42 (55.3)**	9 (11.8)	**19 (40.4)**	18 (38.3)	10 (21.3)	13 (35.1)	**22 (59.5)**	2 (5.4)	0.142
Increase job satisfaction	25 (32.9)	**41 (53.9)**	10 (13.2)	**19 (40.4)**	17 (36.2)	11 (23.4)	14 (37.8)	**22 (59.5)**	1 (2.7)	0.051
Enhances staff knowledge^a^	**50 (66.7)**	21 (28.0)	4 (5.3)	**37 (78.7)**	9 (19.1)	1 (2.1)	**19 (52.8)**	16 (44.4)	1 (2.8)	0.112
**Concerns** (n = 160)										
Work Health and Safety^b^	**43 (55.8)**	29 (37.7)	5 (6.5)	**18 (38.3)**	16 (34.0)	13 (27.7)	**17 (45.9)**	12 (32.4)	8 (21.6)	0.025
Care and handling^b^	**43 (55.8)**	17 (22.1)	17 (22.1)	**18 (38.3)**	11 (23.4)	**18 (38.3)**	**19 (51.4)**	13 (35.1)	5 (13.5)	0.056
Maintenance of sterile field	29 (38.2)	**35 (46.1)**	12 (15.8)	7 (14.9)	17 (36.2)	**23 (48.9)**	10 (27.0)	**23 (62.2)**	4 (10.8)	<0.001
Decrease in direct involvement	30 (39.5)	**34 (44.7)**	12 (15.8)	9 (19.1)	12 (25.5)	**26 (55.3)**	1 (2.7)	**28 (75.7)**	8 (21.6)	<0.001
Increase operation time^c^	33 (42.9)	**36 (46.8)**	8 (10.4)	**38 (80.9)**	7 (14.9)	2 (4.3)	9 (23.7)	**23 (60.5)**	6 (15.8)	<0.001
Space and location^d^	**45 (60.0)**	27 (36.0)	3 (4.0)	15 (31.9)	15 (31.9)	**17 (36.2)**	11 (29.7)	**21 (56.8)**	5 (13.5)	<0.001
Negatively affect team dynamics	11 (14.5)	**45 (59.2)**	20 (26.3)	6 (12.8)	12 (25.5)	**29 (61.7)**	5 (13.5)	**25 (67.6)**	7 (18.9)	<0.001
Add cost and financial pressure	**40 (52.6)**	31 (40.8)	5 (6.6)	**28 (59.6)**	12 (25.5)	7 (14.9)	11 (29.7)	**22 (59.5)**	4 (10.8)	0.018
**Facilitators** (n = 161)										
Theoretical training	**72 (94.7)**	4 (5.3)	0 (0.0)	**43 (93.5)**	2 (4.3)	1 (2.2)	**28 (71.8)**	11 (28.2)	0 (0.0)	0.001
Practical training	**72 (94.7)**	4 (5.3)	0 (0.0)	**45 (97.8)**	1 (2.2)	0 (0.0)	**27 (69.2)**	12 (30.8)	0 (0.0)	<0.001
Educational guides	**69 (90.8)**	7 (9.2)	0 (0.0)	**44 (95.7)**	2 (4.3)	0 (0.0)	**29 (74.4)**	10 (25.6)	0 (0.0)	0.011
Support staff when required	**71 (93.4)**	5 (6.6)	0 (0.0)	**45 (97.8)**	1 (2.2)	0 (0.0)	**28 (71.8)**	11 (28.2)	0 (0.0)	0.001

^a^n = 158

^b^n = 161

^c^n = 162

^d^n = 159.

Medical staff: included consultant surgeon, consultant anaesthetist, fellow, surgical registrar, anaesthetics registrar, resident and intern. Support staff: included radiographer, operations assistant, administrative officer and theatre technician.

### Benefits to staff

The majority of medical staff believed the implementation of RAS will increase the value of staff roles (40.4%) and job satisfaction (40.4%), while nursing and support staff were uncertain about these benefits. All three specialties agreed the implementation of RAS enhances staff knowledge. Although no statistically significant difference was found between the three groups in regards to the benefit of robotic surgery to staff (p>0.05) (Table 2).

### Workplace environment

Most of the nursing, medical and support theatre staff had concerns about care and handling (p = 0.056). Medical staff had concerns that RAS will increase operative time, while nursing and support theatre staff were neutral (p<0.001). The space and location of the robot in the operating theatre was a concern for nursing staff (60.0%), though, medical staff were not concerned (36.2%) and support theatre staff were unsure (56.8%) (p<0.001). Nursing (52.6%) and Medical staff (59.6%) were concerned that RAS will add significant cost and financial pressure on the facility, while support theatre staff were neutral on this point (59.5%) (p = 0.018) (Table 2).

### Facilitators

Most of the nursing, medical and support staff agreed that theoretical, practical training, educational guides and staff support would facilitate the introduction of new technology in the workplace; however, differences were found between groups (Table 2).

## Discussion

This study identifies and describes the knowledge and attitudes of operating theatre staff from a large public tertiary referral hospital in Australia prior to the implementation of RAS.

In terms of RAS knowledge, there was a difference between the staffing groups, with the majority of nursing (58.1%) and support staff (70.3%) reporting no real understanding of the system’s capacity, whilst the majority of medical staff (70.7%) advised they had some or a thorough understanding of RAS (p<0.001). This may be attributed to medical staff having greater exposure to RAS from working in private hospitals. However, most importantly, this finding supports the recognition that different staffing groups may be starting from different knowledge baselines,[6] which should be taken into consideration prior to implementation of an RAS program. The lower levels of RAS knowledge was somewhat surprising given the attention and focus placed on RAS as one of the most innovative surgical technologies today. Nonetheless, it conveys that assumptions regarding staff exposure and knowledge levels about RAS should not be made prior to commencement of a new program.

In terms of skills in robotics, given the survey was undertaken prior to implementation of an RAS program and the limited availability of RAS systems overall across the health sector, it was anticipated the majority of all staff groups would report having no robotic skills. This finding again highlights the importance of taking into consideration the starting point of all staff groups, whereby no prior knowledge or skill level should be expected when planning training and implementation timeframes.

The attitudes of staff towards the implementation of an RAS program was considered across four categories, including benefits to patients, benefits to staff, impact on the workplace environment and key facilitators. Overall, the three staff groups reported predominately neutral attitudes towards the benefits of RAS to patients. The only exception was the majority of medical staff (55.3%) believing that RAS would provide shorter patient length of hospital stay (p<0.001). These results were rather unexpected, given the publicity surrounding the advantages of RAS but seem to indicate that staff reserve their judgement prior to their own experience with RAS. This result may be influenced due to the majority of medical staff (70.7%) reporting some/advanced robotic knowledge when compared to nursing (41.9%) and support staff (29.7%) who reported much less. Furthermore, the medical staff may be more aware of an increasing body of evidence suggesting greater benefits of RAS when compared to other surgical approaches, including open procedures, towards a shorter length of hospital stay.[11] In general, the patterns of responses for all staffing groups were more favourable than negative towards RAS benefiting patients

In terms of benefits to staff, all three staff groups reported that RAS would enhance their knowledge, which may be interrupted as indicating a level of receptiveness to the new technology. The majority of medical staff had a more favourable attitude towards RAS, increasing the value of staff roles (40.4%) and increasing job satisfaction (40.4%) compared to nursing and support staff who reported feeling predominantly neutral on these factors. In general, the patterns of responses were more favourable than negative towards RAS benefiting staff. These results support the previous interpretation that without extensive knowledge and experience of RAS, staff reserve their attitudes about the potential benefits of the system.

Concerns regarding the impact of RAS on the workplace environment were reported by all staff groups for Work Health and Safety, and care and handling. These concerns highlight two areas that should be targeted within an implementation plan to ensure that staff apprehensions are addressed and alleviated prior to commencement of an RAS program. There were mixed views on the other environmental factors across the three staff groups, making it difficult to draw strong conclusions about the overall views of staff. It was anticipated that all staff groups would be worried about the additional cost and financial pressures of implementing an RAS program based on the attention given to this aspect of the technology, however the majority of support staff (59.5%) reported feeling neutral on this issue. The theatres physical space and location seemed to be a concern to nursing staff (60.0%) when compared to medical (31.9%) and support staff (29.7%). This may be due to the layout of our facility that was not originally designed to accommodate RAS with the responsibility of moving the robotic console between different theatres being given to the nurses.

In terms of facilitators for assisting the implementation of an RAS program, all three staff groups overwhelmingly supported the use of all four elements outlined including theoretical and practical training, educational guides and support of staff when required. Whilst these findings don’t highlight any one area of assistance being better received than another, it provides some insight that staff are seemingly receptive to a variety of training and support methods being offered when implementing a new program.

Within our awareness, this is the first study to describe the knowledge and attitudes of theatre staff prior to the implementation of RAS in the public sector. A further strength of this study is the high response rate (over 65%). There were some limitations including potential selection bias as the survey was run over a limited one week period, therefore theatre staff not working on that week missed the opportunity to respond to the survey. The survey was quantitative in nature, rather than being a detailed qualitative investigation of the various factors that impact the knowledge and attitudes of different theatre staff on the implementation of RAS. Furthermore, as the study was only conducted within one facility, the results may not be generalizable to operating theatre staff within other public hospitals across Australia. It is evident future studies addressing these limitations along with the investigation of changes in the knowledge and attitudes of theatre staff after the implementation of RAS within the public sector are warranted.

In conclusion, these results highlight that staffing groups within operating theatres are likely to have different baselines of knowledge and varying attitudes towards the introduction of a complex new intra-operative technology. Whilst in general staff were more favourable towards RAS than negative, they were likely to reserve their judgement prior to their own experiences with implementation of the new robotic system. Concerns regarding Work Health and Safety, and care and handling were conveyed by all staff. Collectively, these findings should be taken into consideration for training and support strategies prior to the implementation of a RAS program.

## Supporting information

S1 Survey RAS survey. (DOCX)
