# Supplementary material for: Knowledge and attitudes of theatre staff prior to the implementation of robotic-assisted surgery in the public sector
Source: PLoS One. 2019 Mar 14;14(3):e0213840. doi: 10.1371/journal.pone.0213840 (PMC6417692; doi:10.1371/journal.pone.0213840)
Supplement: S1 Survey — (DOCX) [file pone.0213840.s001.docx]

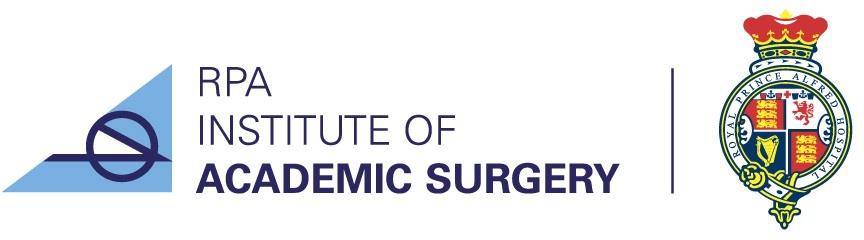


# Staff attitudes

towards SLHD Robotic Surgery Program Survey

**Section 1: This section is to capture the basic demographics of our survey respondants**

## What gender are you

- Male □ Female

## What age bracket are you in

□ 18-34 □ 35-49 □ ≥50

## What is your staff specialty/award

- Nursing all nursing awards)
- Medical Staff (consultant surgeon, consultant anaesthetist, fellow, surgical registrar, anaesthetics registrar, resident and intern)
- Support Staff (radiographer, operations assistant, administrative officer, theatre technician)

## What is your knowledge of the DaVinci XI surgical robot?

- I have never heard of it
- I have some understanding of its capabilities / I have a thorough knowledge of its capabilities

## What is your current skill level in the use of the DaVinci XI robot?

- I have received no training regarding the credentialing requirements of the DaVinci robot/ I have commenced/partially completed training regarding the credentialing requirements of the DaVinci robot
- I have completed basic training regarding the credentialing requirements of the DaVinci robot as per manufacturer requirements/ In addition to basic training I have completed advanced training regarding the credentialing requirements of the DaVinci robot

**Section 2: Based on your current understanding of robotic surgery, please rate your level of agreement with the following statements**

| **6. Benefits of RAS for patients:** | **Disagree** | **Neutral** | **Agree** |
| --- | --- | --- | --- |
| Robotic surgery will help reduce overall length of stay | ⃝ | ⃝ | ⃝ |
| Robotic surgery will reduce patient post-operative pain | ⃝ | ⃝ | ⃝ |
| Robotic surgery will reduce intraoperative complications (e.g. blood loss) compared to current procedures | ⃝ | ⃝ | ⃝ |

| **7. Benefits of RAS for staff:** | **Disagree** | **Neutral** | **Agree** |
| --- | --- | --- | --- |
| Robotic surgery increases the value of staff roles | ⃝ | ⃝ | ⃝ |
| Robotic surgery will increase my job satisfaction | ⃝ | ⃝ | ⃝ |
| Being involved in robotic surgery enhances overall staff knowledge | ⃝ | ⃝ | ⃝ |

| **8. Benefits of RAS for workplace Environment:** | **Disagree** | **Neutral** | **Agree** |
| --- | --- | --- | --- |
| Robotic surgery is a Work Health and Safety (WH&S) concern (additional equipment in the operating theatre) | ⃝ | ⃝ | ⃝ |
| Care and handling of specialised robotic surgery equipment concerns me | ⃝ | ⃝ | ⃝ |
| I am concerned about maintenance of sterile field when assisting in robotic surgery cases more so than when assisting in other cases | ⃝ | ⃝ | ⃝ |
| I am concerned about a decrease in my direct involvement in the case intraoperatively during robotic surgery compared to other procedures | ⃝ | ⃝ | ⃝ |
| I am concerned that robotic surgery will increase operating time | ⃝ | ⃝ | ⃝ |
| I am concerned regarding space and location of the robot | ⃝ | ⃝ | ⃝ |
| I am concerned that robotic surgery will negatively affect current team dynamics in the operating theatre | ⃝ | ⃝ | ⃝ |
| I am concerned that robotic surgery will add significant cost and  financial pressure on our facility | ⃝ | ⃝ | ⃝ |

| **9. Facilitators towards the implementation of new**  **technology:** | **Disagree** | **Neutral** | **Agree** |
| --- | --- | --- | --- |
| Formal, theoretical training | ⃝ | ⃝ | ⃝ |
| Practical training (i.e. simulation) | ⃝ | ⃝ | ⃝ |
| Educational guides and references for use intraoperatively | ⃝ | ⃝ | ⃝ |
| Support staff available when required | ⃝ | ⃝ | ⃝ |
